# Analysis of admixture proportions in seven geographical regions of the state of Guerrero, Mexico

**DOI:** 10.1002/ajhb.23032

**Published:** 2017-07-04

**Authors:** José Ángel Cahua‐Pablo, Miguel Cruz, Pedro Vidal Tello‐Almaguer, Luz Carmen del Alarcón‐Romero, Esteban Juan Parra, Salvador Villerías‐Salinas, Adán Valladares‐Salgado, Vianet Argelia Tello‐Flores, Abigail Méndez‐Palacios, Claudia Paola Pérez‐Macedonio, Eugenia Flores‐Alfaro

**Affiliations:** ^1^ Universidad Autónoma de Guerrero, Facultad de Ciencias Químico Biológicas, Laboratorio de Investigación en Epidemiología Clínica y Molecular Chilpancingo Guerrero 39089 México; ^2^ Instituto Mexicano del Seguro Social, Centro Médico Nacional Siglo XXI, Hospital de Especialidades “Bernardo Sepúlveda”, Unidad de Investigación Médica en Bioquímica Ciudad de México 06725 México; ^3^ Universidad Autónoma de Guerrero, Centro de Investigación y Posgrado en Estudios Socioterritoriales Chilpancingo Guerrero 39020 México; ^4^ Department of Anthropology University of Toronto Mississauga Mississauga Ontario 3359 Canada

## Abstract

**Objective:**

Mexico's current population structure has been defined by admixture between European, Native American, and to some extent African, groups that started in the sixteenth century. The aim of this research was to analyze the relative contributions of these continental population groups to the seven regions of the state of Guerrero, Mexico.

**Methods:**

A total of 104 ancestry informative markers were analyzed in 480 unrelated women from the seven regions of the state of Guerrero. The individual ancestry proportions were estimated using the software ADMIXMAP v3.2.

**Results:**

The relative Native American, European and African ancestral contributions to the whole sample were estimated to be 69%, 27%, and 1.9%, respectively. We observed significant differences in admixture proportions across the regions. The highest average Native American ancestry was found in the Montaña region and the lowest in Costa Grande. Conversely, the highest European contribution was observed in Costa Grande. The highest African contributions were observed in the regions of Costa Chica and Costa Grande.

**Conclusions:**

The genetic structure of the population of Guerrero reflects quite well the historical processes that have occurred in this state. Native American population settlements were mainly in the regions of Montaña, Norte, and Centro, where the highest indigenous genetic contribution is observed today. European settlers came from the center of the state to regions with significant agricultural and mining activities. The highest African contributions are observed in coastal regions, in agreement with historical evidence about slave trade routes in the Americas.

## 1 INTRODUCTION

Hispanic/Latino populations in the Americas exhibit a complex genetic structure which is the result of genetic contributions from Native American, European and African populations. The European component primarily came from settlers from the Iberian Peninsula and southern Europe, and the African component came from persons captured during the slave trade. These historical events have affected patterns of genetic variation in current Hispanic/Latino populations throughout Latin America in a heterogeneous manner (Bryc et al., 2010; Wang et al., 2008).

In Mexico, admixture occurred between numerous indigenous groups inhabiting different regions of the country (Nahua, Otomi, or Maya) and Europeans, mostly Spaniards, who arrived after the conquest by Hernán Cortés in the sixteenth century. Africans, such as Mandingos and Wolofs from West Africa, and Bantu peoples from Central Africa, were transported to Mexico in the 16th‐18th centuries to support the conquest or as slaves (Lalekou, 2016). Today, there are Afro‐descendant populations in different locations of Mexico, in particular the Costa Chica of Guerrero and Oaxaca, in which African heritage is manifested in numerous cultural traditions, including food, music, and traditional medicine (Velázquez & Iturralde, 2012).

Archaeological and linguistic evidence can be used to study these historical processes, but they also have left a signature in the genomes of contemporary populations. Studying this genetic information can contribute to the reconstruction of Mexican population history. Admixture studies are also relevant from the biomedical point of view, as admixture‐based approaches, such as admixture mapping, can identify genetic factors associated with diseases and complex traits (Mao et al., 2007; Shriner et al., 2011). In genetic association studies in admixed populations, individual admixture proportions are routinely incorporated into the statistical models to minimize potential false positives due to population stratification (Flores‐Alfaro, Burguete‐García, & Salazar‐Martínez, 2012).

Guerrero is one of the 32 states in Mexico. It is located in the southern region of the country, bordered on the north by the states of Mexico and Morelos, the northwest by Michoacán, the northeast by Puebla, the east by Oaxaca and the south by the Pacific Ocean. Guerrero is divided into 81 municipalities that comprise seven regions characterized by different cultural, economic, geographic and political traits (Acapulco, Centro, Costa Chica, Costa Grande, Montaña, Norte, and Tierra Caliente).

The demographic characteristics of Guerrero make it an interesting location to conduct admixture studies. It has undergone significant historical events, such as the Spanish conquest and the French intervention, a similar situation that occurred in other southern states of the country and in Central American countries. Before the arrival of the Spaniards to the Americas, Guerrero was inhabited by various indigenous groups, with previous studies showing the proportion of Native American ancestry in Guerrero to be amongst the highest in Mexico (Silva‐Zolezzi et al., 2009).

Although previous admixture studies have reported regional differences in ancestral contributions in Mexico (Silva‐Zolezzi et al., 2009), there have been very few attempts to study regional variation within a single state. Here, we significantly expand previous research by analyzing admixture contributions in women of the seven regions of the state of Guerrero using a panel of autosomal Ancestry Informative Markers (AIMs). Through this approach we attempt to answer two important questions: (1) Are there significant differences in African, European, and Native American admixture proportions between the seven regions? and (2) Do the observed admixture patterns match well known historical population processes within the state of Guerrero? Finally, we compare our results with those described in other regions of Mexico to help elucidate the population history of the country.

## 2 METHODS

### 2.1 Subjects

A total of 480 unrelated women (relatedness was verified during the interview and by their last names) were enrolled in the study. They ranged in age from 30 to 65, and were selected only if they themselves, their parents, and their grandparents were born in the state of Guerrero, Mexico. Women who participated in the study were academic, administrative, or maintenance workers of the Autonomous University of Guerrero (UAGro), and were recruited at their place of work in the different cities of the regions of the state of Guerrero. Those who agreed to participate in the study signed an informed consent prior to providing blood samples to extract DNA. In order to obtain sociodemographic and clinical information, women answered a questionnaire. Pregnant women, or those with a previous diagnosis of cancer, cirrhosis or coronary heart disease, were excluded. The project was approved by the Ethics Committee of the UAGro.

### 2.2 Ancestry informative markers (AIMs)

Ancestry informative markers (AIMs) are genetic loci showing large geographic differentiation that can be used to determine ancestral contributions at the individual and population level (Collins‐Schramm et al., 2004; Tsai et al., 2005). Multiple different panels of AIMs to infer ancestry proportions in recently admixed populations from the Americas have been used (Galanter et al., 2012; Mao et al., 2007; Price et al. 2007; Watkins et al., 2012; Yaeger et al., 2008).

As described elsewhere (Cahua‐Pablo et al., 2015), the AIMs panel used in this study comprises 104 of the 107 highly informative markers described by Yaeger et al. (2008). This panel of markers has been used to characterize admixture proportions in previous studies (Pereira et al., 2012; Risch et al., 2009). Briefly, Yaeger's panel includes biallelic single nucleotide polymorphisms (SNPs) that were selected from the Affymetrix Human 100K SNP chip, based on their information content to infer Native American, European, and African ancestry. The African parental sample comprised 37 individuals from West Africa, the European parental sample consisted of 42 European American samples, and the Native American sample included 15 Maya and 15 Nahua from Mexico. SNPs were selected if the difference in allele frequency (delta value) was at least 50% between pairs of ancestral populations. Detailed information about the parental allele frequencies for each marker is available in Yaeger et al. (2008).

Within the panel of 104 markers used in this study, there are at least 56 markers with frequency differences higher than 50% for each relevant pairwise comparison (African‐European, African‐Native American and European‐Native American). This level of difference minimizes the possibility of biased admixture results. The markers are distributed across the genome, with an average distance between markers of about 2.4 × 10^7^ bp, in order to minimize linkage disequilibrium between markers in the ancestral populations.

Yaeger et al. (2008) selected ∼100 markers based on simulation studies (Yai et al., 2005) showing that this number of AIMs would produce estimates of individual ancestry with *R* ^2^ values >0.9 with respect to the true individual ancestry proportions. In a different study Galanter et al. (2012) showed that panels with ∼100 highly informative AIMs produce individual estimates that are highly correlated with estimates based on dense genome‐wide markers. Given the high information content of the AIMs used in this study, and the selection of parental samples used to identify the AIMs, this set of markers is adequate for the study of geographic patterns of admixture in the state of Guerrero.

### 2.3 DNA extraction and genotyping

From each of the participants, 5 mL of blood was obtained by venipuncture using vacuum extraction tubes with EDTA. Subsequently the leukocytes were separated by centrifugation. Genomic DNA was extracted from leukocytes using the nonenzymatic rapid technique (Lahiri & Nurnberger, 1991), briefly, this method consisted of cell lysis using a low salt buffer containing 10 mM Tris‐HCl pH 7.6, 10 mM KCl, 10 mM MgCl_2_ and 2 mM EDTA (TKM1) and Triton X‐100 a detergent ionic, high salt buffer containing 10 mM Tris‐HCl pH 7.6, 10 mM KCl, 10 mM MgCl_2_, 0.4 M NaCl, and 2 mM EDTA (TKM 2), 10% SDS, add 0.30 mL of 6 M NaCl, and to the supernatant add 2 volumes of 100% ethanol. Concentration and purity were evaluated by spectrophotometry.

AIMs were genotyped at the Biomedical Genomics Center, University of Minnesota, using iPLEX reagents and protocols for multiplex PCR, single base primer extension (SBE) and generation of mass spectra, as per the manufacturer's instructions (Sequenom, San Diego, CA, USA). Multiplexed PCR was performed in 5‐µL reactions on 384‐well plates containing 5 ng of genomic DNA. Reactions contained 0.5U HotStar *Taq* polymerase (QIAGEN Company, Venlo, The Netherlands), 100 nM primers, 1.25× HotStar *Taq* buffer, 1.625 mM MgCl_2_, and 500 µM dNTPs. Following enzyme activation at 94°C for 15 min, DNA was amplified with 45 cycles of 94°C × 20 s, 56°C × 30 s, 72°C × 1 min, followed by a 3‐min extension at 72°C. Unincorporated dNTPs were removed using shrimp alkaline phosphatase (0.3 U, Sequenom). Single‐base extension was carried out by addition of SBE primers at concentrations from 0.625 µM (low MW primers) to 1.25 µM (high MW primers) using iPLEX enzyme and buffers (Sequenom, Inc. John Hopkins Ct, San Diego, CA) in 9‐µL reactions. Reactions were desalted and SBE products measured using the MassARRAY Compact system, and mass spectra analyzed using TYPER software (Sequenom, Inc. John Hopkins Ct, San Diego, CA), in order to generate genotype and allele frequencies.

Each 384‐well plate must have two uniquely‐located, empty (“Blank”) wells, to which nothing had been added. These blanks served a dual purpose. They were negative (no‐template) controls for genotyping assays, and their unique locations served as fingerprints to identify the plate and its orientation. Quality control was performed on all DNA using a two‐part procedure. Quantitative QC (part 1) involved non‐allelic quantitative real‐time PCR using a single TaqMan probe in order to ensure ability to amplify the DNA samples. Qualitative QC (part 2) involved an endpoint reading from a Taqman allelic‐discrimination (SNP) assay that, in addition to providing a second measure of the ability of PCR to amplify each sample, was a sensitive indicator of sample‐to‐sample cross‐contamination, which shows up as dispersed clusters.

### 2.4 Retrieval of information from previous admixture studies in Mexico

Information from previous studies focused on admixture in Mexico was obtained by searching in SCOPUS and PubMed databases from 2000 to 2016. Relevant publications were identified using the following search terms: “Population structure in Mexican‐Mestizos” and “Genetic admixture in Mexico”. These words were combined to retrieve relevant data of admixture in Mexico based on autosomal AIMs. The search also included the review of the bibliography cited at the end of the various research articles. To be selected, the publications had to meet the following criteria: (1) published in peer‐reviewed journals, (2) with independent data, and (3) written in English. We do not include studies based on mtDNA or Y‐chromosome markers, or studies using short tandem repeats, also known as STRs, such as those conducted by Martínez‐Cortés et al. (2012), Rangel‐Villalobos et al. (2008), and Salazar‐Flores et al. (2010).

### 2.5 Statistical analysis

Average admixture proportions, the sum of intensities parameter (equivalent to the average number of generations since the admixture event) and the individual ancestry proportions were estimated using the software ADMIXMAP v3.2 for Windows. We report quantitative data in medians and interquartile range (25th percentile and 75th percentile), or mean and standard deviation, and qualitative data were reported in frequencies. To compare medians and frequencies between regions, Kruskal‐Wallis or Chi‐square (χ^*2*^) tests were used. Variance analysis (ANOVA) followed by the Bonferroni's test was performed to compare means of each ancestral contribution percentage between different regions of the state of Guerrero. A Student's *t*‐test was used to compare mean of autosomal admixture percentage between states in the country. Two‐tailed statistical tests were conducted with a significance level of 5% using STATA v.13 software.

## 3 RESULTS

Women who participated in the study were recruited in different cities in the state of Guerrero (Acapulco, Chilpancingo, Taxco, Iguala, Chilapa, Ometepec, etc.). Regardless of the place of recruitment, they were stratified by region based on the birthplace of their grandparents (Acapulco, Centro, Costa Chica, Costa Grande, Montaña, Norte, and Tierra Caliente).

Part of the analysis of the women entailed determining anthropometric and clinical characteristics, and the presence of diseases. This information is presented in Table 1. The median age of women was 46 years and the median body mass index (BMI) was 27 kg/m^2^. No significant differences in age or BMI were observed between regions, which indicates that despite different sample sizes and stratification by region, there is little evidence of selection bias at least in terms of health statistics. This is also supported by the lack of differences in the frequency of diabetes and metabolic syndrome among regions. Otherwise, it was found that body height and years of schooling were significantly lower in the Montaña region, an area that has been historically marginalized due to its geographical location. We consider it important to show in Table 1 the characteristics of the studied population to show that there is no selection bias.

**Table 1:** Sociodemographic and clinical characteristics, and admixture proportion of the studied women

		Region							
Characteristic	Total*n* = 480	A *n* = 24	C *n* = 178	CC *n* = 58	CG *n* = 58	M *n* = 32	N *n* = 79	TC *n* = 51	*P* value
Age (years)	46 (38–53)	39 (33–47)	46 (38–53)	47 (37–53)	49 (41–52)	44 (36–50)	47 (40–55)	43 (37–51)	0.108*
Height (m)	1.55 (1.5–1.58)	1.55 (1.53–1.6)	1.54 (1.5–1.58)	1.54 (1.51–1.57)	1.56 (1.53–1.6)	1.48 (1.4–1.53)	1.55 (1.51–1.58)	1.56 (1.53–1.6)	<0.001*
Weight (kg)	65 (59–74)	65 (59–75)	66 (58–74)	65 (61–71)	70 (61–78)	60 (52–68)	65 (58–75)	64 (60–76)	0.073*
BMI (kg/m^2^)	27 (25–30)	26 (24–29)	28 (25–31)	27 (26–30)	28 (25–31)	27 (24–30)	27 (25–31)	26 (24–30)	0.788*
Schooling (years)	17 (12–18)	17 (15–19)	17 (12–17)	17 (15–19)	17 (17–19)	12 (8–17)	17 (17–19)	17 (12–17)	<0.001*
Diabetes, *n* (%)									
No	438 (91)	23 (96)	163 (92)	53 (91)	52 (90)	29 (91)	73 (92)	45 (88)	0.995†
Yes	42 (9)	1 (4)	15 (8)	5 (9)	6 (10)	3 (9)	6 (8)	6 (12)	
SM, *n* (%)									
No	317 (66)	19 (79)	118 (66)	42 (72)	32 (55)	25 (78)	53 (67)	28 (55)	0.092†
Yes	163 (34)	5 (21)	60 (34)	16 (28)	26 (45)	7 (22)	26 (33)	23 (45)	
**Admixture proportion (%), mean ± standard deviation**									
NA	69.2 ± 13.5	69.2 ± 14.7	70.1 ± 13.0	66.4 ± 13.3	61.4 ± 13.1	79.9 ± 11.7	72.3 ± 12.2	66.7 ± 12.2	**<0.001**‡
European	27.0 ± 12.1	27.2 ± 13.6	27.4 ± 12.3	25.7 ± 11.1	31.6 ± 11.3	18.2 ± 10.7	25.6 ± 11.9	29.7 ± 11.2	**<0.001**‡
African	3.7 ± 4.7	3.6 ± 4.2	2.5 ± 2.6	7.9 ± 7.7	7.0 ± 5.7	1.9 ± 1.9	2.0 ± 1.9	3.6 ± 4.2	**<0.001**‡

Data are reported as medians (25th–75th percentile) or as noted in table. *Kruskal‐Wallis test; ^†^Chi‐square test; ^‡^analysis of variance (ANOVA).

A: Acapulco; C: Centro; CC: Costa Chica; CG: Costa Grande; M: Montaña; N: Norte; TC: Tierra Caliente; BMI: body mass index; HT: hypertension; NA: Native American.

**Figure 1:**
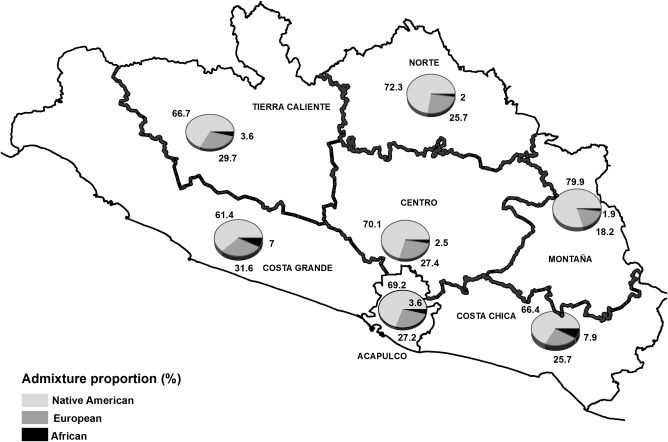
The average admixture proportions (%) for each region of the state of Guerrero are shown. The highest Native American admixture proportions are found in the Montaña region (79.9%), while the highest European proportion is observed in Costa Grande (31.6%). African ancestry is relatively low, but higher African contributions are observed on both Coasts of the State, relative to the interior

We found significant differences in the ancestral proportions observed in different regions of the state of Guerrero. Native American ancestry was highest in Montaña (79.9%) and lowest in the Costa Grande (61.4%), while European ancestry was highest in Costa Grande (31.6%) and lowest in Montaña (18.2%). African ancestry was higher on both coasts of Guerrero compared to other regions, as seen in Costa Chica with 7.9% and Costa Grande with 7.0% (Figure 1). Native American ancestry was significantly higher in the Montaña region compared to other regions [Centro (*p* = .002), Costa Chica (*p* < .001), Costa Grande (*p* < .001), and Tierra Caliente (*p* < .001)]. The proportion of European ancestry was significantly lower in Montaña compared to the regions: Centro (*p* = .001), Costa Grande (*p* < .001), and Tierra Caliente (*p* < .001). On the other hand, African ancestry was significantly higher in both coasts compared to Acapulco, Centro, Montaña, Norte, and Tierra Caliente (*p* < .001) (Supporting Information Table S1).

Native American genetic ancestry was significantly higher in the state of Guerrero compared to the states of Sonora, Guanajuato, Veracruz, and Yucatan (*p* < .001), and was similar to that seen in Mexico City. On the other hand, European ancestry was significantly higher in the state of Sonora compared with other states (*p* < .001). Moreover, in the state of Guerrero, African ancestry was higher compared to other states, although not so with Mexico City (Supporting Information Table S2).

## 4 DISCUSSION

Genetic studies that have been carried out in Mexico to date indicate a broad range of admixture proportions across the country (Table 2). In the most comprehensive admixture study, Silva‐Zolezzi et al. (2009) used a set of 1,814 AIMs to study admixture proportions in different regions of Mexico. They observed average Native American, European, and African admixture proportions in the global sample of 55.2%, 41.8% and 1.8%, respectively. However, there was considerable geographic variability in admixture proportions. The highest Native American admixture proportions were found in the states of Guerrero (66.0%) and Yucatan (58.8%), and the lowest in Sonora (36.2%). Conversely, the highest European admixture proportions were observed in Sonora (61.6%) and the lowest in Guerrero (28.5%). Silva‐Zolezzi et al. (2009) suggested that these patterns are due to the unique demographic and historic conditions of the different regions within Mexico. In particular, the geographic patterns observed in Native American contributions are correlated with the population density (both pre‐ and postcontact) of indigenous groups in different regions of Mexico. African ancestry was, in general, very low throughout the country (<5%), with the highest estimate being reported for Guerrero (4.1%), and the lowest for Yucatan (0.8%).

**Table 2:** Autosomal admixture percentage reported for different regions of Mexico

			Percentage of ancestral contribution			
State or city	*N*	AIMs	Native American	European	African	References
México (Country), mean ± SD	300	1814	55.2 ± 15.4	41.8 ± 15.5	1.8 ± 3.5	Silva‐Zolezzi et al. (2009)
Sonora	ns	1814	36.2 ± 8.9	61.6 ± 8.5	1.2 ± 1.7	Silva‐Zolezzi et al. (2009)
Zacatecas, mean ± SD	ns	1814	51.1 ± 77	45.7 ± 8.4	1.8 ± 2.3	Silva‐Zolezzi et al. (2009)
Nuevo León, mean (rank)	100	74	56 (27.4–81.2)	38 (16.7–70.5)	6 (1.3–11.9)	Martínez‐Fierro et al. (2009)
Guanajuato, mean ± SD	Ns	1814	57.6 ± 9.6	39.9 ± 10	1.1 ± 1.8	Silva‐Zolezzi et al. (2009)
México City	561	69	65	30	5	Martínez‐Marignac et al. (2007)
México City	429	10	69.9	25.1	4.97	Villarreal‐Molina et al. (2007)
México City	378	15	69	26	5	Juárez‐Cedillo et al. (2008)
México City	1310	446	64.2	32.4	3.5	Galanter et al. (2012)
Veracruz, mean ± SD	ns	1814	61.3 ± 14.1	35.6 ± 13	2 ± 4.2	Silva‐Zolezzi et al. (2009)
Guerrero, mean ± SD	ns	1814	66 ± 13.8	28.5 ± 12	4.1 ± 6.1	Silva‐Zolezzi et al. (2009)
Guerrero, mean ± SD	480	104	69.2 ± 13.5	27.0 ± 12.1	3.7 ± 4.7	Our study, 2017
Yucatán, mean ± SD	ns	1814	58.8 ± 16.1	39.2 ± 16.2	0.8 ± 1.2	Silva‐Zolezzi et al. (2009)

SD: standard deviation; ns: not specified.

Several studies in Mexico City (Galanter et al., 2012; Juárez‐Cedillo et al., 2008; Martinez‐Marignac et al., 2007; Villarreal‐Molina et al., 2007) have reported concordant admixture estimates for this area, with Native American ancestry ranging between 64.2% and 69.9%, European ancestry between 25.1% and 32.4%, and African ancestry between 3.5% and 5%. Montinaro et al. (2015) also reported admixture proportions in a sample from Mexico, which was primarily characterized by admixture between European (47.6%) and East Asian/American groups (49.8%), with a small African contribution (2.6%).

The state of Guerrero is ethnically, culturally and geographically diverse. We conducted a study in women originating from different geographic regions in order to determine in detail ancestry proportions throughout the state (Figure 1). We found an average Native American contribution of 69%, with the lowest proportions observed in Costa Grande with 61.4% and the highest in Montaña with 79.9%. The average European ancestral contribution in the state was estimated as 27%, and was lowest in Montaña (18.2%) and highest in Costa Grande (31.6%). The African contribution was substantially lower than the Native American and European contributions, with a mean of 3.7%. However, there are clear geographic patterns in the distribution of African ancestry within Guerrero, with higher proportions in the coastal areas than in the interior.

Our study indicates that the major genetic contribution to the current population of Guerrero comes from indigenous groups. Based on our findings, the highest indigenous contribution in Guerrero is observed in the regions of Montaña (79.9%), Centro (70.1%), and Norte (72.3%). These are the highest Native American admixture proportions reported to date in Mexico (see Table 2). Upon the arrival of Spaniards to Guerrero, several indigenous communities lived in this region. Barlow (cited by Commons 2003) reported that the lordship of Yopitzingo was situated in the Central‐South region of the state and the Purépechas or Tarascos were located in the northwest of the state (Commons, 2003). There are a number of indigenous communities currently living in Guerrero, including the Amuzgos (Ñom daa), Náhuatl (Naua), Mixtecos (Ñuu Savi) and Tlapanecos (Me'phaa) (Estado de Guerrero, México, 2011).

**Figure 2:**
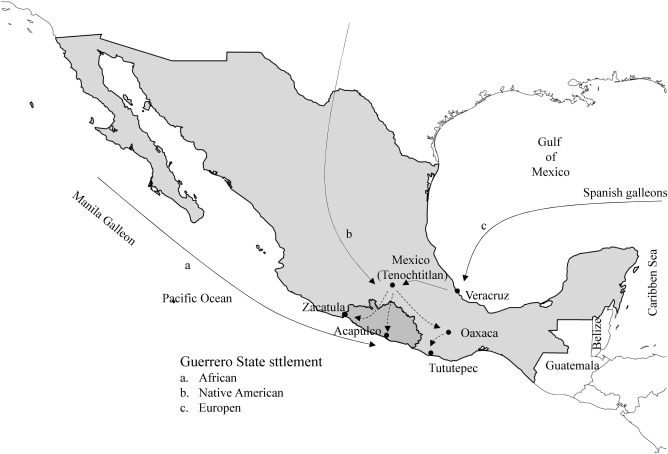
The process of settlement of the state of Guerrero is indicated. When the Europeans arrived, there were several indigenous communities living in the region. Europeans arrived primarily from the Atlantic Ocean, initially Spanish settlers and later also French and Belgians. African slaves primarily arrived to the state from the Pacific

The Spanish settlement occurred practically in all of the regions that currently make up the state of Guerrero (Figure 2). Expeditions were initially directed to strategic areas of the territory (Zacatula, Acapulco, and Tututepec). The main objective of these expeditions was to displace the native inhabitants for military, religious, and colonization purposes, to establish Spanish dominion, and take hold of large areas of terrain in order to exploit natural resources (García‐Castro, 2011). In addition to the arrival of people from Spain, some sources have indicated that in the Norte and Sierra regions there were also French and Belgians who were part of the French army and settled in the region of Tierra Caliente (Ruiz 1896). In our study, the highest European ancestry is observed in the regions of Costa Grande (31.6%), Tierra Caliente (29.7%), and Centro (27.4%).

The presence of individuals of African origin in the state of Guerrero dates back to the early years of Spanish colonization in Mexico. Historical reports described that, in 1527, there were several black slaves in the coastal region of Guerrero. In the first two villas that were founded in the Coast of Guerrero—San Luis Acatlán in Costa Chica (1522), and the Concepción in Zacatula of Costa Grande (1523)—Spanish encomenderos had African slaves despite prohibitions by the King. Most of the enslaved Africans were transferred to Acapulco via the route Veracruz‐Mexico‐Acapulco. These slaves were dedicated to the work of caulking boats, as well as other tasks (Enciclopedia Guerrerense, 2012; Illades, 2000).

Another route of arrival for individuals of African descent in the Costa Chica region was the Veracruz‐Puebla‐Oaxaca route; Pinotepa del Rey (today Pinotepa Nacional) and Cortijos were the locations that received the first slaves arriving by this route. These slaves engaged in cattle farming, pearl diving, gold mining in rivers and streams, and salt production. Huatulco was an important place in the settlement of individuals of African descent along the Pacific coast. To a similar degree, the Cimarrons (eg, escaped enslaved Africans) populated this region in the late sixteenth century (Enciclopedia Guerrerense, 2012; Velázquez & Iturralde, 2012).

As described above, in their admixture analysis Silva‐Zolezzi et al. (2009) reported the highest African ancestry in the state of Guerrero (4.1%). Our study provides additional insights about African ancestry in this state. We show that, consistent with historical evidence, the highest African ancestry occurs in both coasts of the state (7.9% in Costa Chica and 7.0% in Costa Grande), and the lowest in the Montaña region (1.9%).

In summary, we report here the most extensive analysis of admixture in the State of Guerrero carried out to date. Although the sample size of our study is limited, and samples were only collected from women, we have been able to explore in detail the admixture proportions in seven different regions of Guerrero. The main patterns observed in our analysis correspond quite well with historical population settlement processes. Indigenous settlements were mainly located in Centro, Montaña, and Norte, where we also see the highest estimates of indigenous ancestry today. European settlers came from the state's center to the regions where the development of agricultural and mining activities was feasible. Initially, this took place in flat areas near rivers or streams, which allowed the establishment of farms, and mining activities had their greatest splendor in the city of Taxco. In contrast to the Native American and European ancestry proportions, the average African ancestry proportions that we estimated in our sample from Guerrero are relatively low, possibly because the economy did not demand significant slave labor, due to its geographic and orographic features.

Future studies using genome‐wide data will make it possible to obtain more detailed insights on the admixture history of the state of Guerrero. These data, in combination with other dense datasets available for the relevant parental groups, will provide information about many aspects of population history that cannot be explored with a limited panel of AIMs, such as the timing and number of pulses of migration, potential sex‐biases in gene flow, and the origin of the European settlers and enslaved Africans that have contributed to the current population of Guerrero.

## CONFLICT OF INTEREST

The authors declare that they have no competing interests.

## Supporting information

Supporting Information S1

## References

[ajhb23032-bib-0001] Bryc, K. , Velez, C. , Karafet, T. , Moreno‐Estrada, A. , Reynolds, A. , Auton, A. , … Ostrer, H. (2010). Colloquium paper: Genome‐wide patterns of population structure and admixture among Hispanic/Latino populations. Proceedings of the National Academy of Sciences of the United States of America, 107, 8954–8961. 20445096 10.1073/pnas.0914618107 PMC3024022

[ajhb23032-bib-0002] Cahua‐Pablo, J. Á. , Cruz, M. , Méndez‐Palacios, A. , Antúnez‐Ortiz, D. L. , Vences‐Velázquez, A. , Alarcón‐Romero, L. del, C. , … Flores‐Alfaro, E. (2015). Polymorphisms in the LPL and CETP genes and haplotype in the ESR1 gene are associated with metabolic syndrome in women from Southwestern Mexico. International Journal of Molecular Sciences, 16, 21539–21554. 26370976 10.3390/ijms160921539 PMC4613266

[ajhb23032-bib-0003] Collins‐Schramm, H. E. , Chima, B. , Morii, T. , Wah, K. , Figueroa, Y. , Criswell, L. A. , … Seldin, M. F. (2004). Mexican American ancestry‐informative markers: examination of population structure and marker characteristics in European Americans, Mexican Americans, Amerindians and Asians. Human Genetics, 114, 263–271. 14628215 10.1007/s00439-003-1058-6

[ajhb23032-bib-0004] Commons A. Gestación y nacimiento de un estado: Guerrero . (2003). *Investigaciones Geográficas, Boletín del Instituto de Geografía*, UNAM, 50, 196–219.

[ajhb23032-bib-0005] Enciclopedia Guerrerense: Negritud en Guerrero . (2012). *Guerrero Cultural Siglo XXI, A. C* http://www.enciclopediagro.org/index.php/indices. Accessed August 6, 2016.

[ajhb23032-bib-0006] Estado de Guerrero, México . (2011). *Ley número 701 de reconocimiento, derechos y cultura de los pueblos y comunidades indígenas del estado de Guerrero* http://i.administracion2014-2015.guerrero.gob.mx/uploads/2011/06/L701RDCPCIEG1.pdf. Accessed August 14, 2016.

[ajhb23032-bib-0007] Flores‐Alfaro, E. , Burguete‐García, A. I. , & Salazar‐Martínez, E. (2012). Genetic epidemiology research designs. Revista Panamericana De Salud Pública, 31, 88–94. 22427170 10.1590/s1020-49892012000100013

[ajhb23032-bib-1008] Galanter, J. M. , Fernandez‐Lopez, J. C. , Gignoux, C. R. , Barnholtz‐Sloan, J. , Fernandez‐Rozadilla, C. , Via, M. , … Carracedo A. ; LACE Consortium. (2012). Development of a panel of genome‐wide ancestry informative markers to study admixture throughout the Americas. PloS Genetics, 8, e1002554. doi: 10.1371/journal.pgen. 22412386 10.1371/journal.pgen.1002554 PMC3297575

[ajhb23032-bib-0009] García‐Castro, N. (2011). *Los grados de asimilación económica del estado de Guerrero, a fines del siglo XX* Doctoral thesis. FFyL‐UNAM. Available http://www.remeri.org.mx/portal/REMERI.jsp?id=oai:tesis.dgbiblio.unam.mx:000678290. Accessed September 20, 2016.

[ajhb23032-bib-0010] Illades, C. (2000). Breve historia de Guerrero. México: Fondo de Cultura Económica.

[ajhb23032-bib-0011] Juárez‐Cedillo, T. , Zuñiga, J. , Acuña‐Alonzo, V. , Pérez‐Hernández, N. , Rodríguez‐Pérez, J. M. , Barquera, R. , … Vargas‐Alarcón, G. (2008). Genetic admixture and diversity estimations in the Mexican Mestizo population from Mexico City using 15 STR polymorphic markers. Forensic Science International Genetics, 2, 37–39. 10.1016/j.fsigen.2007.08.017 19083813

[ajhb23032-bib-0012] Lahiri, D. K. , & Nurnberger, J. I., Jr. (1991). A rapid non‐enzymatic method for the preparation of HMW DNA from blood for RFLP studies. Nucleic Acids Research, 19, 5444. 1681511 10.1093/nar/19.19.5444 PMC328920

[ajhb23032-bib-0013] Lalekou, K. L. (2016). Los negros y la construcción de la nación mexicana. Humania Del Sur: Revista De Estudios Latinoamericanos, Africanos y Asiáticos, 11(20), 139–154.

[ajhb23032-bib-0014] Mao, X. , Bigham, A. W. , Mei, R. , Gutierrez, G. , Weiss, K. M. , Brutsaert, T. D. , … Parra, E. J. (2007). A genomewide admixture mapping panel for Hispanic/Latino populations. American Journal of Human Genetics, 80, 1171–1178. 17503334 10.1086/518564 PMC1867104

[ajhb23032-bib-0015] Martínez‐Cortés, G. , Salazar‐Flores, J. , Fernández‐Rodríguez, L. G. , Rubi‐Castellanos, R. , Rodríguez‐Loya, C. , Velarde‐Félix, J. S. , … Rangel‐Villalobos, H. (2012). Admixture and population structure in Mexican‐Mestizos based on paternal lineages. Journal of Human Genetics, 57, 568–574. 22832385 10.1038/jhg.2012.67

[ajhb23032-bib-0016] Martinez‐Fierro, M. L. , Beuten, J. , Leach, R. J. , Parra, E. J. , Cruz‐Lopez, M. , Rangel‐Villalobos, H. , … Rojas‐Martinez, A. (2009). Ancestry informative markers and admixture proportions in northeastern Mexico. Journal of Human Genetics, 54, 504–509. 19680268 10.1038/jhg.2009.65

[ajhb23032-bib-0017] Martinez‐Marignac, V. L. , Valladares, A. , Cameron, E. , Chan, A. , Perera, A. , Globus‐Goldberg, R. , … Parra, E. J. (2007). Admixture in Mexico City: Implications for admixture mapping of type 2 diabetes genetic risk factors. Human Genetics, 120, 807–819. 17066296 10.1007/s00439-006-0273-3

[ajhb23032-bib-0018] Montinaro, F. , Busby, G. B. , Pascali, V. L. , Myers, S. , Hellenthal, G. , & Capelli, C. (2015). Unravelling the hidden ancestry of American admixed populations. Nature Communications, 24, 1–7. 10.1038/ncomms7596 PMC4374169 25803618

[ajhb23032-bib-0019] Pereira, L. , Zamudio, R. , Soares‐Souza, G. , Herrera, P. , Cabrera, L. , Hooper, C. C. , … Tarazona‐Santos, E. (2012). Socioeconomic and nutritional factors account for the association of gastric cancer with Amerindian ancestry in a Latin American admixed population. PLoS One, 7, e41200. 22870209 10.1371/journal.pone.0041200 PMC3411699

[ajhb23032-bib-0020] Price, A. L. , Patterson, N. , Yu, F. , Cox, D. R. , Waliszewska, A. , McDonald, G. J. , … Reich, D. (2007). A genomewide admixture map for Latino populations. American Journal of Human Genetics, 80, 1024–1036. 17503322 10.1086/518313 PMC1867092

[ajhb23032-bib-0021] Rangel‐Villalobos, H. , Muñoz‐Valle, J. F. , González‐Martín, A. , Gorostiza, A. , Magaña, M. T. , & Páez‐Riberos, L. A. (2008). Genetic admixture, relatedness, and structure patterns among Mexican populations revealed by the Y‐chromosome. American Journal of Physical Anthropology, 135, 448–461. 18161845 10.1002/ajpa.20765

[ajhb23032-bib-0022] Risch, N. , Choudhry, S. , Via, M. , Basu, A. , Sebro, R. , Eng, C. , … Gonzalez‐Burchard, E. (2009). Ancestry‐related assortative mating in Latino populations. Genome Biology, 10, R132. 19930545 10.1186/gb-2009-10-11-r132 PMC3091325

[ajhb23032-bib-0023] Ruíz, E. (1896). *Historia de la Guerra de Intervención en Michoacán* México: Secretaría de Fomento. https://archive.org/details/historiadelague00ruizgoog. Accessed August 01, 2016.

[ajhb23032-bib-0024] Salazar‐Flores, J. , Dondiego‐Aldape, R. , Rubi‐Castellanos, R. , Anaya‐Palafox, M. , Nuño‐Arana, I. , Canseco‐Avila, L. M. , … Rangel‐Villalobos, H. (2010). Population structure and paternal admixture landscape on present‐day Mexican‐Mestizos revealed by Y‐STR haplotypes. American Journal Human Biology, 2, 401–409. 10.1002/ajhb.21013 19967759

[ajhb23032-bib-0025] Shriner, D. , Adeyemo, A. , Ramos, E. , Chen, G. , & Rotimi, C. N. (2011). Mapping of disease‐associated variants in admixed populations. Genome Biology, 12, 223. 21635713 10.1186/gb-2011-12-5-223 PMC3219963

[ajhb23032-bib-0026] Silva‐Zolezzi, I. , Hidalgo‐Miranda, A. , Estrada‐Gil, J. , Fernandez‐Lopez, J. C. , Uribe‐Figueroa, L. , Contreras, A. , … Jimenez‐Sanchez, G. (2009). Analysis of genomic diversity in Mexican Mestizo populations to develop genomic medicine in Mexico. Proceedings of the National Academy of Sciences of the United States of America, 106, 8611–8616. 19433783 10.1073/pnas.0903045106 PMC2680428

[ajhb23032-bib-0027] Tsai, H. J. , Choudhry, S. , Naqvi, M. , Rodriguez‐Cintron, W. , Burchard, E. G. , & Ziv, E. (2005). Comparison of three methods to estimate genetic ancestry and control for stratification in genetic association studies among admixed populations. Human Genetics, 118, 424–433. 16208514 10.1007/s00439-005-0067-z

[ajhb23032-bib-0028] Velázquez, M. E. , & Iturralde, G. (2012). Afrodescendientes en México. Una historia de silencio y discriminación. México: Conapred, Instituto Nacional de Antropología e Historia.

[ajhb23032-bib-0029] Villarreal‐Molina, M. T. , Aguilar‐Salinas, C. A. , Rodríguez‐Cruz, M. , Riaño, D. , Villalobos‐Comparan, M. , Coral‐Vazquez, R. , … Romero‐Hidalgo, S. (2007). The ATP‐binding cassette transporter A1 R230C variant affects HDL cholesterol levels and BMI in the Mexican population: Association with obesity and obesity‐related comorbidities. Diabetes, 56, 1881–1887. 17287470 10.2337/db06-0905

[ajhb23032-bib-0030] Wang, S. , Ray, N. , Rojas, W. , Parra, M. V. , Bedoya, G. , Gallo, C. , … Ruiz‐Linares, A. (2008). Geographic patterns of genome admixture in Latin American Mestizos. PLoS Genetics, 4, e1000037. doi: 10.1371/journal.pgen. 18369456 10.1371/journal.pgen.1000037 PMC2265669

[ajhb23032-bib-0031] Watkins, W. S. , Xing, J. , Huff, C. , Witherspoon, D. J. , Zhang, Y. , Perego, U. A. , … Jorde, L. B. (2012). Genetic analysis of ancestry, admixture and selection in Bolivian and Totonac populations of the New World. BMC Genetics, 20, 13:39. 10.1186/1471-2156-13-39 PMC3432609 22606979

[ajhb23032-bib-0032] Yaeger, R. , Avila‐Bront, A. , Abdul, K. , Nolan, P. C. , Grann, V. R. , Birchette, M. G. , … Joe, A. K. (2008). Comparing genetic ancestry and self‐described race in African Americans born in the United States and in Africa. Cancer Epidemiology, Biomarkers, & Prevention, 17, 1329–1338. 10.1158/1055-9965.EPI-07-2505 PMC2507870 18559547

