# Supplementary material for: Analysis of admixture proportions in seven geographical regions of the state of Guerrero, Mexico
Source: Am J Hum Biol. 2017 Jul 4;29(6):e23032. doi: 10.1002/ajhb.23032 (PMC5697696; doi:10.1002/ajhb.23032)
Supplement: Supplementary file 1 — Supporting Information S1 [file AJHB-29-na-s001.docx]

**Table S1- Comparison individual admixture percentage between regions of the state of Guerrero**

| Region | **Native American** | | | | | | **European** | | | | | | **African** | | | | | |
| --- | --- | --- | --- | --- | --- | --- | --- | --- | --- | --- | --- | --- | --- | --- | --- | --- | --- | --- |
|  | A | C | CC | CG | M | N | A | C | CC | CG | M | N | A | C | CC | CG | M | N |
| C | 1.0 |  |  |  |  |  | 1.0 |  |  |  |  |  | 1.0 |  |  |  |  |  |
| CC | 1.0 | 1.0 |  |  |  |  | 1.0 | 1.0 |  |  |  |  | **0.001** | **<0.001** |  |  |  |  |
| CG | 0.258 | **<0.001** | 0.762 |  |  |  | 1.0 | 0.424 | 0.164 |  |  |  | **0.015** | **<0.001** | 1.0 |  |  |  |
| M | 0.046 | **0.002** | **<0.001** | **<0.001** |  |  | 0.105 | **0.001** | 0.082 | **<0.001** |  |  | 1.0 | 1.0 | **<0.001** | **<0.001** |  |  |
| N | 1.0 | 1.0 | 0.165 | **<0.001** | 0.107 |  | 1.0 | 1.0 | 1.0 | 0.081 | 0.057 |  | 1.0 | 1.0 | **<0.001** | **<0.001** | 1.0 |  |
| TC | 1.0 | 1.0 | 1.0 | 0.699 | **<0.001** | 0.299 | 1.0 | 1.0 | 1.0 | 1.0 | **<0.001** | 1.0 | 1.0 | 1.0 | **<0.001** | **<0.001** | 1.0 | 0.700 |
| A: Acapulco; C: Centro; CC: Costa Chica; CG: Costa Grande; M: Montaña; N: Norte; TC: Tierra Caliente  Data represent p-values obtained by the Bonferroni’s post hoc test.  In bold the significant differences of the mean of the ancestral proportions between regions | | | | | | | | | | | | | | | | | | |

**Table S2- Comparison of autosomal admixture percentage among the States of México**

| State/Region | Native American | | | | | | European | | | | | | African | | | | | |
| --- | --- | --- | --- | --- | --- | --- | --- | --- | --- | --- | --- | --- | --- | --- | --- | --- | --- | --- |
|  | SR | ZS* | GT | CX* | VZ | GR | SR | ZS | GT | CX* | VZ | GR | SR | ZS | GT* | CX* | VZ | GR |
| SR/Noroeste |  |  |  |  |  |  |  |  |  |  |  |  |  |  |  |  |  |  |
| ZS/Norte* | 0.180 |  |  |  |  |  | **<0.001** |  |  |  |  |  | 0.142* |  |  |  |  |  |
| GT/Centro | **<0.001** | 0.556 |  |  |  |  | **<0.001** | **0.002** |  |  |  |  | 0.776 | 0.093 |  |  |  |  |
| CX/Centro | **<0.001** | 0.208 | **0.001** |  |  |  | **<0.001** | **<0.001** | **<0.001*** |  |  |  | **<0.001*** | **<0.001*** | **<0.001** |  |  |  |
| VZ/Golfo | **<0.001** | 0.361 | 0.128 | **0.001** |  |  | **<0.001*** | **<0.001*** | 0.067 | **0.006** |  |  | 0.216 | 0.769* | 0.168 | **0.016** |  |  |
| GR/Sur | **<0.001** | 0.103 | **<0.001** | 0.843 | **<0.001** |  | **<0.001*** | **<0.001*** | **<0.001** | **<0.001** | **<0.001** |  | **<0.001*** | **<0.001*** | **<0.001** | 0.433 | **0.014** |  |
| YN/Sureste | **<0.001** | 0.492 | 0.652 | **<0.001** | 0.411 | **<0.001** | **<0.001*** | **0.014*** | 0.796 | **<0.001** | 0.223 | **<0.001** | 0.178 | **0.008*** | 0.330 | **<0.001** | 0.057* | **<0.001*** |
| Data represent p-values obtained by t-student test. *Unequal variances  SR: Sonora; ZS: Zacatecas; GT: Guanajuato; CX: Ciudad de México; VZ: Veracruz; GR: Guerrero; YN: Yucatán | | | | | | | | | | | | | | | | | | |
